# Atypical Chédiak-Higashi syndrome with attenuated phenotype: three adult siblings homozygous for a novel *LYST* deletion and with neurodegenerative disease

**DOI:** 10.1186/1750-1172-8-46

**Published:** 2013-03-22

**Authors:** James D Weisfeld-Adams, Lakshmi Mehta, Janet C Rucker, Francine R Dembitzer, Arnold Szporn, Fred D Lublin, Wendy J Introne, Vikas Bhambhani, Michael C Chicka, Catherine Cho

**Affiliations:** 1 Department of Genetics & Genomic Sciences, Mount Sinai School of Medicine, New York, NY, USA; 2 Department of Neurology, Mount Sinai School of Medicine, New York, NY, USA; 3 Department of Ophthalmology, Mount Sinai School of Medicine, New York, NY, USA; 4 Department of Pathology, Mount Sinai School of Medicine, New York, NY, USA; 5 Section on Human Biochemical Genetics, National Institute of Child Health and Human Development, National Institutes of Health, Bethesda, MD, USA; 6 Prevention Genetics, Marshfield, WI, USA

**Keywords:** Chédiak-Higashi syndrome, LYST, Lysosomal, Amyloid, Oxidative stress, Parkinsonism, Neurodegenerative disease

## Abstract

**Background:**

Mutations in *LYST*, a gene encoding a putative lysosomal trafficking protein, cause Chédiak-Higashi syndrome (CHS), an autosomal recessive disorder typically characterized by infantile-onset hemophagocytic syndrome and immunodeficiency, and oculocutaneous albinism. A small number of reports of rare, attenuated forms of CHS exist, with affected individuals exhibiting progressive neurodegenerative disease beginning in early adulthood with cognitive decline, parkinsonism, features of spinocerebellar degeneration, and peripheral neuropathy, as well as subtle pigmentary abnormalities and subclinical or absent immune dysfunction.

**Methods:**

In a consanguineous Pakistani kindred with clinical phenotypes consistent with attenuated CHS, we performed SNP array-based homozygosity mapping and whole gene sequencing of *LYST*.

**Results:**

We identified three individuals homozygous for a novel six base pair in-frame deletion in *LYST* (c.9827_9832ATACAA), predicting the loss of asparagine and threonine residues from the LYST transcript (p.Asn3276_Thr3277del), and segregating with the phenotype in this family.

**Conclusions:**

We further characterize the neurologic features of the attenuated form of CHS, and discuss pathophysiologic mechanisms underlying the neurodegenerative components of CHS. Attenuated CHS is phenotypically heterogenous and should be considered when young adults develop neurodegenerative disease and have pigmentary abnormalities. We briefly discuss surveillance and management of patients with CHS-related neurodegeneration.

## Background

Mutations in *LYST* result in Chédiak-Higashi syndrome (CHS; MIM 214500), a rare autosomal recessive disorder typically characterized by immune dysfunction in early childhood, partial oculocutaneous albinism and other pigmentary abnormalities, and, in survivors of the accelerated phase of immune deterioration, late-onset neurologic dysfunction. Progressive neurodegenerative disease with subtle pigmentary abnormalities and absent or subclinical immunologic abnormalities are the hallmarks of the attenuated form of CHS [1-3]. This form of CHS has been estimated to account for 10–15% of all CHS patients [4], but may be underdiagnosed. Neurologic manifestations appear to be variable, but frequently include peripheral neuropathy, intellectual decline, parkinsonism, ataxia, and white matter abnormalities [4-8]. Genotyping of individuals with these phenotypes has, to date, identified only four causative *LYST* genotypes, all of them missense mutations; infantile-onset disease is associated with null *LYST* alleles. Here we report a large consanguineous family with three adult siblings each homozygous for a novel deletion in exon 43 of *LYST* and with variable pigmentary abnormalities and adult-onset neurodegenerative disease.

## Methods

### Patients

**Figure 1:**
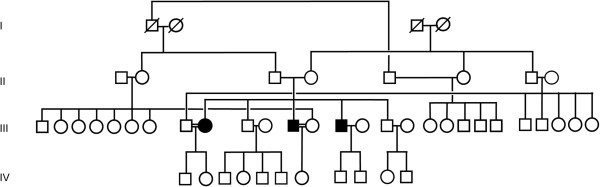
**Family pedigree.** Shaded shapes indicate affected individuals. The proband and Brother 1 (III:9 and III:12, respectively) are both married to their first cousins. The spouses of Brother 2 (III:14), and the two unaffected brothers (III:10 and III:16) are from families unrelated to this sibship.

For participation and publication of this material, written consent was obtained from all participants in accordance with the policies and procedures of the institutional ethics committee of the Mount Sinai School of Medicine, and in compliance with the Helsinki Declaration. In the three affected patients (III:9, female, 43 years; III-12, male, 38 years; III-14, male, 33 years; see Figure 1), the diagnosis was established by medical history and detailed clinical evaluation. Neurologic testing encompassed standard testing of gait, balance, and coordination, magnetic resonance imaging (MRI) of the brain and, in the case of III-14, of the spine, as well as electromyography (EMG) and nerve conduction studies. Ophthalmologic examination consisted of dilated funduscopy, Goldmann perimetry and standard tests of color and contrast sensitivity. Immunologic testing employed natural killer (NK) cell functional assays and immunoglobulin testing. Peripheral blood smears were visualized using Wright-Giemsa staining and oil immersion. Hair shafts were analyzed using standard light microscopy. Skin biopsy samples were analyzed using standard hematoxylin and eosin and Congo Red staining, and immunohistochemistry.

Genetic diagnosis was confirmed with sequencing of the entire coding region of *LYST* in the proband. Blood samples were obtained for DNA extraction, and genomic DNA was isolated using standard protocols. Samples for each patient were received at Prevention Genetics as DNA. Exonic and immediate flanking DNA of the *LYST* gene was amplified using standard PCR conditions and KAPATaq HotStart enzyme (Kapa Biosystems, Woburn, MA, USA). PCR primers were tailed with m13 forward and reverse priming sequences. PCR products were sequenced in both forward and reverse directions using m13 primers and BigDye dideoxynucleotide terminators (Applied Bio, Carlsbad, CA, USA). Patient DNA sequences were analyzed using SeqScape software (v2.5, Applied Bio, Carlsbad, CA, USA).

## Results

### Clinical evaluation

Three affected siblings were part of a large consanguineous family of Pakistani extraction (Figure 1). The proband (III:9), was 40 years old at the time of initial evaluation in Medical Genetics. She had a 9 year history of progressive leg weakness with cognitive impairment, cerebellar ataxia with hypermetric saccades, and parkinsonian features including bradykinesia, masked facies, and hypophonia. In childhood, she had a history of learning difficulties and attention-deficit behaviors without hyperactivity. She had a long history of arthralgias and limb contractures. She had generalized hyperpigmentation, noted in infancy and followed by the appearance of widespread hypopigmented macules persisting into adulthood. She had a history of multiple dental infections in childhood and severe periodontitis at the age of 36 years, but no other history suggestive of immunodeficiency. There was no history of bleeding. Physical examination revealed generalized hyperpigmentation with widespread small hypopigmented macules and patches distributed over her entire body. She had age-appropriate hair graying. She was wheelchair-bound, and exhibited bradykinesia, hypomimia, hypophonia, pseudobulbar affect, cerebellar ataxia, ocular dysmetria, spasticity and motor weakness with near plegia in the lower limbs. Deep tendon reflexes were reduced in the lower limbs but hyperactive in the upper limbs. Neuropsychologic testing revealed a mild dementia syndrome. Neuro-ophthalmologic evaluation demonstrated decreased color vision and contrast sensitivity. There was myopic astigmatism bilaterally. There was severe optic nerve pallor and peripheral patchy retinal hypopigmentation bilaterally. Goldmann perimetry revealed central loss. Parkinsonian symptoms did not improve with levodopa-carbidopa, and she experienced lightheadedness.

**Figure 2:**
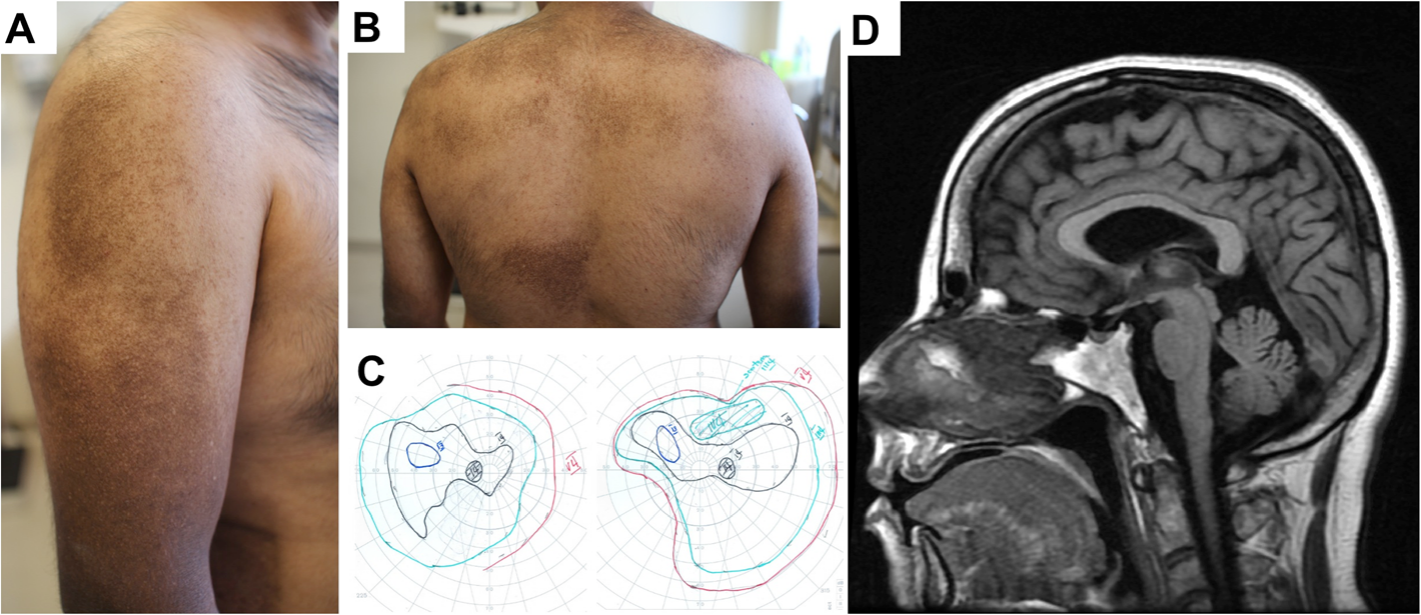
**(A) and (B) Macroscopic skin appearances of Brother 2 at age 33 years.** He reported hair graying dating from adolescence. He recalled abnormal skin pigmentation appearing in early adulthood; this is in contrast to his sister, whose parents report had developed pigmentary abnormalities in early childhood. Her skin changed in appearance over time, initially exhibiting generalized hyperpigmentation, but with development of hypopigmented macules in early adulthood. (**C**) Goldmann visual fields of Brother 1 at aged 38 years. Despite significant central and peripheral field loss, he reported no subjective deficits in peripheral vision. His affected sister and brother also were found to have central field loss on visual field testing. Although retinal appearances were grossly normal, retinal visualization with ocular coherence tomography (OCT) showed nerve fiber thinning to varying extents in the three affected siblings. (**D**) Sagittal T1 MRI study the brain of the female proband at 40 years of age showing mild cerebral and cerebellar volume loss. Subtle white matter signal abnormality in periventricular white matter was also seen on T2/FLAIR sequences (not shown).

Two younger brothers had similar cutaneous appearances and extracutaneous symptoms to their sister (Figure 2A and B). Neither was reported to have a history of infections, headaches, or other neurologic symptoms during childhood, or hospitalizations. Brother 1 (III:12), developed hand incoordination and tremor early in his fourth decade, and, later, gait impairment. Examination demonstrated leg weakness with cognitive impairment, cerebellar ataxia, and parkinsonian features including mild rigidity, bradykinesia, masked facies, and hypophonia. Gait showed reduced stride length and reduced arm swing bilaterally. Deep tendon reflexes were symmetrically reduced. Similar to the proband, he had learning disability and attention-deficit without hyperactivity during childhood. A trial of levodopa-carbidopa initially provided subjective benefit, but caused light-headedness and was later discontinued. Examination did not change. Selegiline reportedly improved the patient’s activity levels and caused a subjective reduction in tremor. Trihexyphenidyl resulted in subjective improvements in rigidity, bradykinesia, and sialorrhea, although physical examination remained essentially unchanged. There was no graying of the hair. Funduscopy revealed mild optic nerve pallor, right congenital hypertrophy of retinal pigment epithelium and left choroidal nevus. Visual acuity was normal, but contrast sensitivity was reduced. Goldmann perimetry revealed central and peripheral loss (Figure 2C).

Brother 2 (III:14) has residual voiding difficulties and right lower extremity pain after having been diagnosed with radiculopathy secondary to a spinal arteriovenous malformation (AVM) aged 28 years. He developed bilateral, symmetric, progressive leg weakness age 29, a year after embolization of his AVM. He reported graying from his teenage years. He developed upper extremity tremor, worse on the right side, at the age of 31. On most recent physical evaluation, at age 33, he had brisk reflexes and weakness in lower extremities, with normal neuropsychologic testing, generally scoring in the low average to average range. Greatest neuropsychologic deficits were in finger dexterity, confrontation naming, and executive functioning. He had diffuse pigmentary abnormalities, especially apparent on the limbs and back, and similar in appearance to those seen in his brother and sister. Funduscopy was normal, although contrast sensitivity was mildly reduced. Goldmann perimetry revealed central loss. He was given a trial of levodopa-carbidopa for parkinsonian symptoms, but did not tolerate it. Neither affected brother had symptoms or signs suggestive of immunodeficiency or abnormal bleeding, although Brother 1 did exhibit subclinical evidence of periodontitis.

### Neuroimaging and neurophysiology

Serial MRI of the proband’s brain (performed at aged 37 years, 40 years, and 43 years) revealed global atrophy (prominence of CSF spaces; volume loss of cerebellar vermis), and diffuse, subtle increases in T2/FLAIR signal intensity in periventricular white matter (Figure 2D) becoming more prominent over time. Brother 1 also had mild global atrophy on MRI at 38 years with mild cerebellar volume loss and a similar subtle T2/FLAIR pattern to his sister. Brother 2 presented with progressive paraparesis 12 months after embolization of the spinal AVM, at which time MRI failed to demonstrate progression or expansion of the AVM; MRI brain at aged 33 years was reported as normal. Electromyography (EMG) and nerve conduction studies in the proband demonstrated reduced amplitudes with normal conduction velocities and left peroneal nerve F waves, consistent with subacute axonal neuropathy, more severe in the lower limb. Brother 1 had EMG changes consistent with a chronic neurogenic process with apparent motor neuropathy, worse distally. Brother 2 had no electrophysiologic evidence of peripheral neuropathy with results suggestive of polyradiculopathy.

### Tissue sampling and immunologic studies

**Figure 3:**
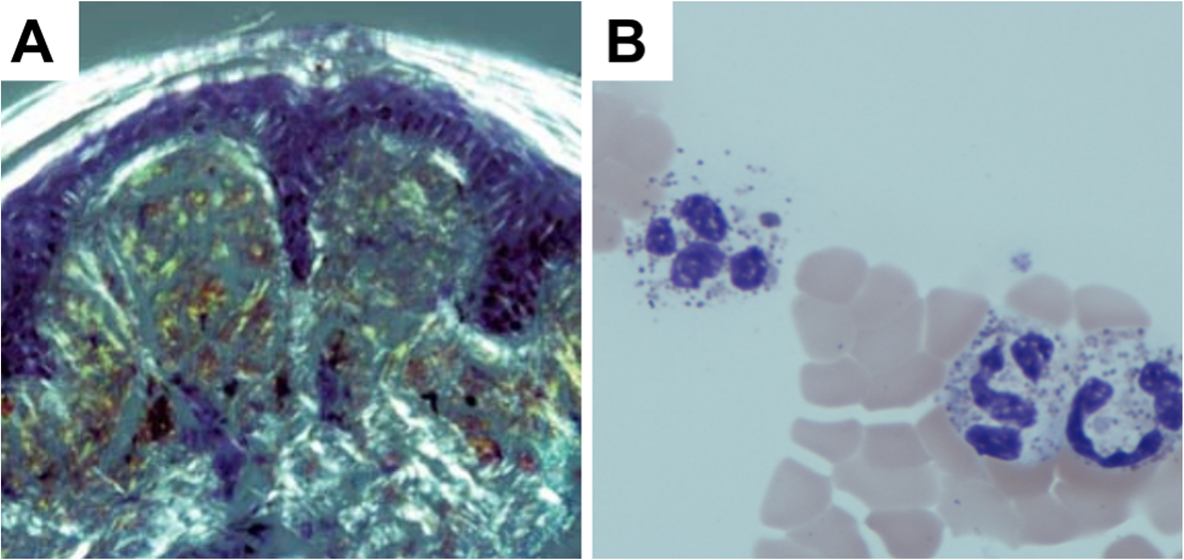
**(A) In a skin biopsy sample from the proband, keratinocytic origin of amyloid was demonstrated by immunohistochemistry for high molecular weight cytokeratin, which showed extensive deposition of amyloid in the papillary and superficial reticular dermis.** The dermal deposits showed positivity with Congo red staining and apple green birefringence was apparent in the expanded papillary dermis with polarization. Additionally, fissured amphophilic globules were observed, containing scattered melanophages and extending into the superficial reticular dermis (not shown). (**B**) Peripheral blood smear demonstrating polymorphonuclear leukocytes with enlarged, variably-sized cytoplasmic granules (Wright Giemsa, oil immersion).

Skin biopsy on the proband demonstrated appearances suggestive of amyloidosis cutis dyschromica (ACD) with extensive deposition of amyloid which appeared to be of keratinocytic origin (Figure 3A). All three affected siblings had normal complete blood count indices, coagulation studies, liver function, and lipid profiles. Peripheral smear in all three siblings demonstrated abnormal polymorphonuclear leukocytes with numerous enlarged, variably-sized cytoplasmic granules (Figure 3B). The proband and both brothers had pigmentary clumping evident on light microcopy of hair shafts. Fibrinogen levels at baseline were high normal or mildly elevated, and ferritin levels were in the low normal range. Natural killer cell function was absent or very low (0.0–0.6 lytic units; normal >2.6) in all three siblings. Quantitative IgG, IgA and IgM were within normal limits.

### Genetic testing

**Figure 4:**
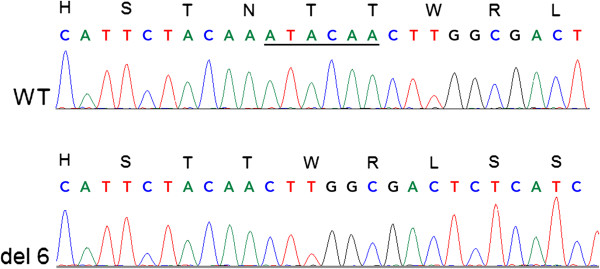
**Sequence chromatogram comparing wild type *LYST *sequence (top row) to sequence from proband, who was found to be homozygous for a pathogenic deletion in exon 43 of *LYST *(c.9827_9832ATACAA, bottom row).** Homozygosity for this deletion was also confirmed in both clinically affected brothers. Both parents (II:3 and II:4) and both clinically unaffected brothers (III:10 and III:16) were confirmed as heterozygotes for the deletion.

Single nucleotide polymorphism (SNP) array on genomic DNA from the proband did not show any significantly increased regions of homozygosity. Sequencing of the entire coding region of *LYST* identified homozygosity for a novel six base pair in-frame deletion in exon 43 (c.9827_9832ATACAA, Figure 4), predicting the loss of asparagine and threonine residues from LYST (p.Asn3276_Thr3277del). The proband was also homozygous for an intronic sequence variant (c.10701 + 8C>T) distal to the deletion. Homozygosity for the same pathogenic deletion was confirmed in both clinically affected brothers. The parents (II:3 and II:4) and both apparently unaffected brothers (III:10 and III:16) were confirmed as heterozygotes for the deletion. The two elder affected siblings’ apparently unaffected partners (III:8 and III:13) are also undergoing testing given their status as first cousins of their spouses; neither of the spouses, nor any of the three children of these two couples has any clinical features of CHS.

### Other studies

The proband had appearances suggestive of hepatic steatosis on abdominal ultrasound; neither brother had this finding. All three siblings had normal splenic and renal appearances on abdominal ultrasound. Of interest, both affected brothers had low levels of total testosterone (205 and 155 ng/dL respectively; normal 260–1080).

## Discussion

We present three adult siblings as examples of a rare, attenuated form of Chédiak-Higashi syndrome, manifesting with progressive neurodegenerative disease and with mild or subclinical immunologic abnormalities, and abnormal pigmentation. Neurologic manifestations in this form of CHS are variable, but frequently include peripheral neuropathy, cognitive decline, and features of spinocerebellar degeneration with cerebral and cerebellar volume loss and abnormal white matter appearances on MRI [4,7,8]. Dystonia has also been reported [9]. HLA-identical allogeneic bone marrow transplant (BMT) was reported as an acceptable curative treatment for classic CHS almost 20 years ago [10]. Despite demonstrating efficacy in treating the immunodeficiency component of the disease, similar neurodegenerative features have also been observed at a similar age in BMT recipients with classical CHS phenotypes [11,12]. Our patients had learning difficulties, attention-deficit behaviors and other neuropsychologic deficits that may have significantly predated the onset of frank neurologic symptoms. Classical CHS has also been associated with learning difficulties in young children, and, later, with progressive, diffuse white matter abnormalities on MRI as well as neurologic decline and accelerated immune deterioration [4,13,14].

*LYST* is a ubiquitously expressed gene whose protein product is concerned with control of exocytosis and trafficking from secretory lysosomes [3,5,15,16] and may be critical to microtubular stability. Several distinct domains within the LYST polypeptide appear to be critical to protein-protein interactions and organization of protein complexes important to lysosome-related organelles [17]. Cytoplasmic giant granules, seen in leucocytes, are a hallmark of CHS, and are also seen in neuronal cells and perineuronal tissue [3]. Enlargements of lysosome-derived organelles are also characteristically observed [18]. Consistent with our experience, other authors [8,19] have reported that granules in patients with attenuated disease are enlarged, but not as dramatically as is seen in classic forms of the disease; it is therefore conceivable that the diagnosis may be missed by inexperienced technologists. With regard to observed neurologic deterioration with advancing age, clumped irregular melanin granules have been observed in the substantia nigra in human patients at autopsy [20,21]. Murine models of CHS with homozygous missense mutations in *LYST* have exhibited predominant neurologic phenotypes including lower motor performance scores than controls, with accumulation of giant lysosomes in neuronal cells and intracytoplasmic inclusions in Purkinje cells of the cerebellum and motor cortex [22]. At autopsy, adults with CHS had neuronal degeneration involving the inferior olivary nuclei and the cerebellar cortex, with an overall distribution similar to late cortical cerebellar atrophy (LCCA) and olivopontocerebellar atrophy (OPCA), but without pontine involvement [20].

AA protein (reactive) amyloidosis is usually observed in the setting of chronic infectious or inflammatory disease. Systemic AA amyloidosis has been reported previously in the setting of CHS, both in humans [20] and in animals [18]. The only previous human report described a Japanese woman with classic CHS who suffered recurrent pyogenic infections and albinism noted from infancy, and had post-mortem evidence of amyloid deposition in multiple tissues including the kidney, liver, thyroid, and ovary, and pituitary and parotid glands. Reactive gliosis was observed in the brain. The authors speculated that recurrent infections had contributed to the development of amyloidosis, and that amyloid deposition would become an increasingly common observation in CHS patients with classic phenotypes as a function of cumulative burden of recurrent, severe infection, especially as these patients survive into adolescence and adulthood in greater numbers as is anticipated as BMT protocols become more refined with improved efficacy and reduced toxicity [20]. Neutropenia, observed chronically or intermittently, has been reported in CHS [23], and the fact that our patients were not neutropenic at the time of evaluation does not exclude historical episodes of neutropenia. However, in view of the fact that our patients do not have any significant history of recurrent infection, but have skin biopsy appearances consistent with amyloid deposition, we suggest that amyloidosis may be a component of the pathophysiology of CHS that is independent of the burden of recurrent infection.

It has been suggested that neurologic manifestations result directly from defective LYST in neurons and glial cells, or from lymphocytic tissue infiltration during the accelerated phase of the disease. Acute CNS manifestations, as well as the presence of long-term neurologic defects, are described in other inherited hemophagocytic disorders (Griscelli syndrome, X-linked lymphoproliferative disease). Each of these disorders is amenable to BMT [24], but neurologic disease develops despite successful BMT. Like CHS, Griscelli syndrome type 2 can cause pigmentary abnormalities with hair graying, as well as neurological disease, and is an important differential diagnosis in this setting. Expanding knowledge of the role of lymphocytic CNS infiltration in causing neurodegenerative disease in CHS may determine optimal timing of transplant in CHS patients, given the fact that many patients, if not all, appear destined to develop debilitating neurodegenerative disease over the longer term. In the United States, transplant if routinely offered in advance of the accelerated phase of classic disease. In a single-center review of the use of hematopoietic stem cell transplantation (HSCT) in Griscelli syndrome type 2 [25], many patients continued to suffer major neurological deficits after HSCT, leading the authors to suggest that transplanting these patients prior to the accelerated phase could be beneficial. The presence of neurodegenerative signs appearing in classical CHS patients many years after BMT suggests that neurodegeneration may be related in part to defective lysosomal trafficking and vacuolar dysfunction. More recently, it has been shown in a murine model that various pathophysiologic aspects of CHS might be directly dependent on oxidative membrane damage [17]. Due to the large size of neurons and their expansive plasma membranes, these and other cell types may be especially sensitive to the adverse effects of accumulation of oxidatively damaged cell membranes.

The in-frame nature of the novel *LYST* deletion, present in the homozygous state in all of the affected siblings in this family, may account for the comparatively mild, late-onset phenotype in these individuals. The deletion is located distally in *LYST* (exon 43 out of 51), and causes loss of two amino acid residues in the BEACH domain of LYST, but does not cause a frameshift/premature termination. To our knowledge, only four other mutations, all missense, have been definitively associated with attenuated phenotypes. A previously reported patient with classic CHS [12] was a compound heterozygote for single base pair deletion (c.9893delT) in a similar region of the BEACH domain and a more proximal nonsense mutation (c.1540C>T). The deletion caused a frameshift resulting in premature protein truncation downstream (F3298fsX3304), and resulted in an infantile-onset immunodeficiency and lymphoproliferative histiocytosis phenotype. Homozygosity for the mutant allele in our cases suggests the siblings’ parents share a common ancestor. Parents denied being directly related, although both families originate from the same region of Pakistan. CHS has not, to our knowledge, been previously reported in Pakistani kindreds.

Based on our experiences, and the available literature [8,9,26], patients with this pattern of CHS might benefit, at least in the short term, from L-dopa, selegiline, trihexylphenidyl, biperiden, or amantadine. At diagnosis, patients require multidisciplinary input from a neurologist with expertise in movement disorders, as well as a clinical immunologist (even if historical clues suggesting immunodeficiency are absent), an ophthalmologist, a neurophysiologist, and a neuroradiologist. Both affected brothers in this family had below normal levels of testosterone but had fathered children; the significance of this finding is unclear and requires further study.

## Conclusions

This family expands the phenotype of CHS, and provides additional characterization of central nervous system disease in such patients. These patients have complex medical needs and require carefully integrated multidisciplinary care. The pathophysiology of this form of the condition is likely to be related to a combination of factors including microtubular instability, impaired lysosomal trafficking, oxidative injury, and amyloid deposition. Variant CHS is phenotypically heterogenous and should be considered when young adults develop neurodegenerative disease in association with pigmentary abnormalities. The disorder can be easily screened for with characteristic findings on peripheral blood smear and hair sampling, although these may be less conspicuous than in classic CHS phenotypes.

## Competing interests

The authors declare that they have no competing interests.

## Authors’ contributions

JWA and LM performed initial evaluation of the patient, made the clinical diagnosis, orchestrated the multidisciplinary evaluation, and wrote the manuscript. JCR oversaw the patients’ ophthalmologic evaluation and care. FRD and AS were responsible for pathologic analysis of tissue samples and blood smears. FDL assisted with neurologic evaluation. The patients saw WJI and VB at an independent clinical evaluation. MCC performed initial whole gene sequencing of *LYST* on the proband, and targeted sequencing for the disease-causing deletion in other affected and unaffected family members. CC oversaw the patients’ neurological evaluation and care and co-authored the manuscript. All authors read, edited, and approved the final version of the manuscript.
